# Characterizing the spatial distributions of spotted lanternfly (Hemiptera: Fulgoridae) in Pennsylvania vineyards

**DOI:** 10.1038/s41598-020-77461-9

**Published:** 2020-11-25

**Authors:** Ashley Leach, Heather Leach

**Affiliations:** 1 Entomology Department, Purdue University, West Lafayette, IN USA; 2 Entomology Department, Penn State University, University Park, PA USA

**Keywords:** Agroecology, Invasive species

## Abstract

Spotted lanternfly (SLF) is an invasive insect in the Northeastern U.S. projected to spread nationally and globally. While SLF is a significant pest of vineyards, little is known about the pest in grape agroecosystems including its spatial ecology. SLF spatial patterns were analyzed using a combination of approaches including generalized linear mixed effect models, Moran’s I statistic for spatial clustering, and Empirical Bayesian Kriging. Analysis revealed that SLF displayed significantly clumped distributions in monitored vineyards. Approximately 54% and 44% of the respective adult and egg mass populations were observed within the first 15 m of the vineyard edge. Importantly, the spatial concentration of adults at the edge was consistent temporally, both between years and weeks. Moreover, high populations of SLF on vines were significantly correlated with reduced fruit production in the following year. Mark-release-recapture of SLF revealed that higher proportions of SLF were recaptured on vines with high pre-existing SLF populations, indicating that SLF may exhibit aggregation behavior along vineyard perimeters. Monitoring and management efforts for SLF should be prioritized around vineyard edges as it may significantly reduce infestations and subsequent damage.

## Introduction

The spatial ecology of invasive species has received considerable attention in the past decade^1–6^. Spatial ecology offers unique insights into how organisms organize and utilize habitats across both space and time. Geostatistical and spatial analyses identify predictable invasion patterns of invasive pests, which aid in describing how these species colonize foreign habitats and what landscape attributes contribute to the their success^7,8^. Importantly, the use of spatial ecology in pest management can identify vulnerable ecosystems (agricultural and otherwise) as well as inherent weaknesses in the pest’s ecology which may be exploited in management efforts.

Spotted lanternfly (SLF), *Lycorma delicatula* (White) (Hemiptera: Fulgoridae), is a new invasive insect in the Northeastern United States native to China^9^. Prior to SLF’s introduction to the US, it has become invasive in Korea and Japan^10,11^. Owing to its broad host range, high fecundity, and inconspicuous egg masses, SLF is expected to invade regions throughout North America and into new countries^12,13^. SLF has been reported to feed on 70 different plant hosts, including many valuable ornamental and agricultural crops^9^. Damage caused by SLF and corresponding efforts to offset feeding damage are expected to cost millions in the United States. Recent economic analyses from Pennsylvania indicate that SLF infestations have already amounted to an annual economic loss of nearly 50.1 million dollars (USD) statewide^14^.

Grape production is greatly impacted by SLF infestations. In the Northeast, growers report reduced vine production or complete plant loss with poor SLF control. Numbers of SLF on individual vines can easily exceed 400 adults, with seasonal averages of 7.5 SLF per vine^15^. SLF adults and nymphs feed on plant phloem from the trunk, cordon, or shoot tissue^15^ which likely reduces photosynthetic potential and vigor of vines. Damage caused by these insects may significantly alter grape production. In a recent 2020 survey, most grape growers (53%) stated that they would forgo replanting or expanding their production without effective SLF management options (H. Leach, unpublished). The need for effective management strategies is paramount to securing the livelihoods and industries impacted by SLF.

Some studies have identified approaches to managing SLF infestations including applications of entomopathogens^16^ and agrichemicals^17^, predation from natural enemies^18^, and chipping wood that contains egg masses^19^. Currently, pest management for SLF in grape has relied heavily on repeated insecticide applications. Unsurprisingly, the number of insecticide applications in SLF infested vineyards has increased drastically, with most vineyards increasing applications by 2–5 times as compared to pre-SLF infestation^20^. SLF is expected to continue to be a serious threat to many agricultural and ornamental industries. However, the success of SLF management tactics relies on appropriate timing and placement. Therefore, it is critical that research addresses not only effective management options but also the biology underpinning the success of these management tactics.

Management of SLF could significantly benefit from an improved understanding of the spatial patterns and damage caused by this pest. Scouting and monitoring can be targeted to specific problem areas, and insecticide use tailored to areas where infestations are highest. Despite a number of recent studies examining basic biology, management tactics, and monitoring efforts^21–23^, no studies have identified the geostatistical or spatial patterns of SLF in grape production. The objectives of this study were to characterize SLF adult and egg distributions across space and time within commercial vineyards in Pennsylvania. Using a combination of weekly monitoring data and grid sampling from 6 to 9 Pennsylvania vineyards, we examined the spatiotemporal relationships of SLF in grape agroecosystems. Mark-recapture of adult SLF was also used to evaluate dispersal behavior based on existing abundance of SLF within vineyards. Further, vine health metrics were measured to determine associations with SLF infestation. We hypothesized that SLF would aggregate around vineyard edges, with greatest densities of SLF adults and egg masses within the perimeter of vineyards. We also predicted that SLF would be negatively associated with vine health.

## Results

### Weekly monitoring

#### Adults

**Figure 1:**
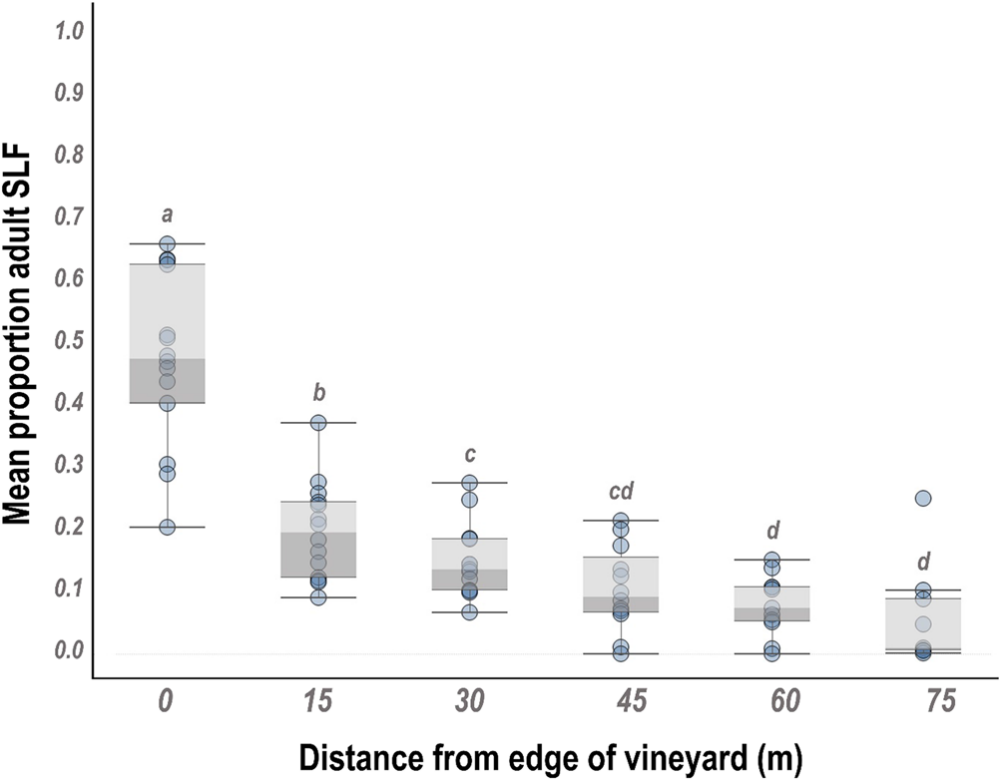
Mean proportion adult spotted lanternfly (SLF) found at sampling distances from vineyard edge, 0 m (at vineyard edge) to 75 m into the vineyard block. A total of 8 vineyards were sampled for adult SLF during 2018 and 2019. Bands for each distance represent 95% confidence interval. Different letters denote significant differences between distances at alpha = 0.05.

SLF adults exhibited strong edge effects in all vineyards sampled (χ^2^ = 237.3, df = 5, p < 0.0001). Approximately, 54% of SLF were found within the first 15 m of the vineyard edge (Fig. 1). Proportions of SLF decreased as distance from the vineyard edge increased. Similar patterns were observed throughout the growing season in both sampling years (Supplemental Fig. S1).

At vineyards where both hybrid and *V. vinifera* vines were monitored (4/8 vineyards), SLF distribution was significantly impacted by the interaction between grape variety and distance from vineyard edge (χ^2^ = 29.1 df = 5, p < 0.0001). Greater proportions of SLF were observed within the first two distances on hybrid vines (Supplemental Fig. S2a, distances 0 m and 15 m) as compared to *V. vinifera* vines where the first distance had greater proportions than all subsequent distances. Generally, larger vineyard blocks had a greater edge effect compared to smaller blocks (χ^2^ = 16.2, df = 5, p = 0.006). However, the pattern was similar for both small (> 0.5 ha) and large (> 1.5 ha) vineyard blocks, and SLF proportions decreased from the vineyard edge. (Supplemental Fig. S2b).

#### Egg masses

**Figure 2:**
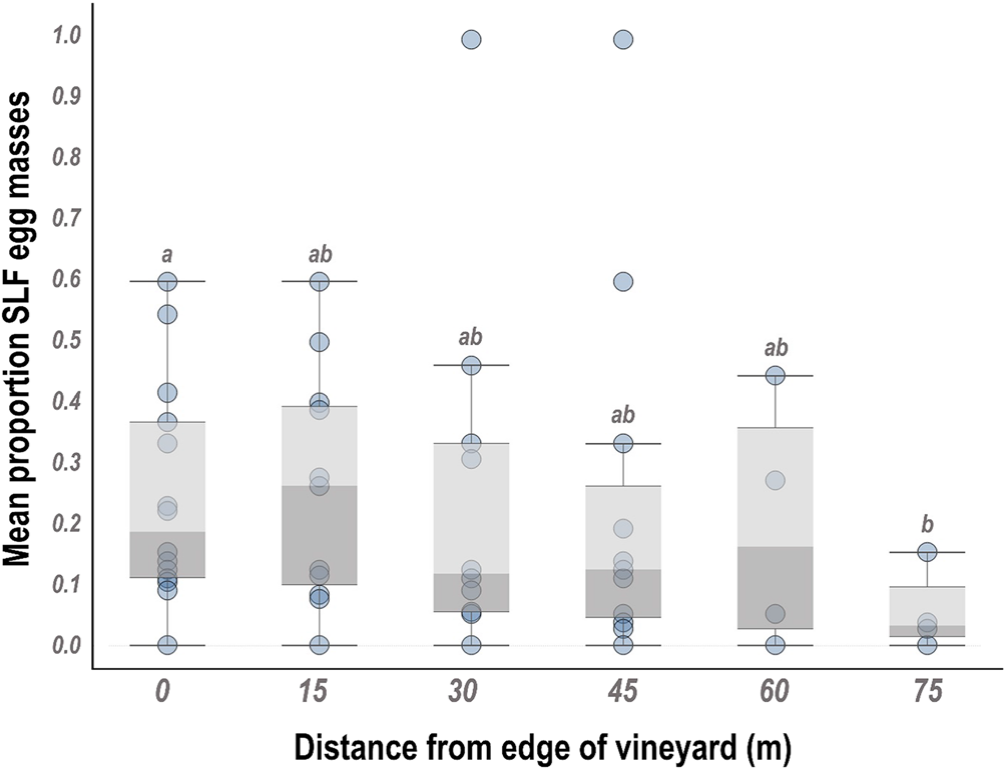
Mean proportion spotted lanternfly (SLF) egg masses found sampling distances from vineyard edge, 0 m (at vineyard edge) to 75 m into the vineyard block. A total of 8 vineyards were sampled for SLF egg masses during 2018 and 2019. Bands for each distance represent 95% confidence interval. Different letters denote significant differences between distances at alpha = 0.05.

The number of egg masses deposited significantly decreased with distance from the vineyard edge (χ^2^ = 11.1, df = 4, p = 0.02). Similar to the findings reported for adults, 44% of SLF egg masses were found within 15 m of the vineyard edge and a significantly lower proportions of egg masses (12%) were found on vines located 75 m from the vineyard block edge (Fig. 2).

#### Vine health

**Figure 3:**
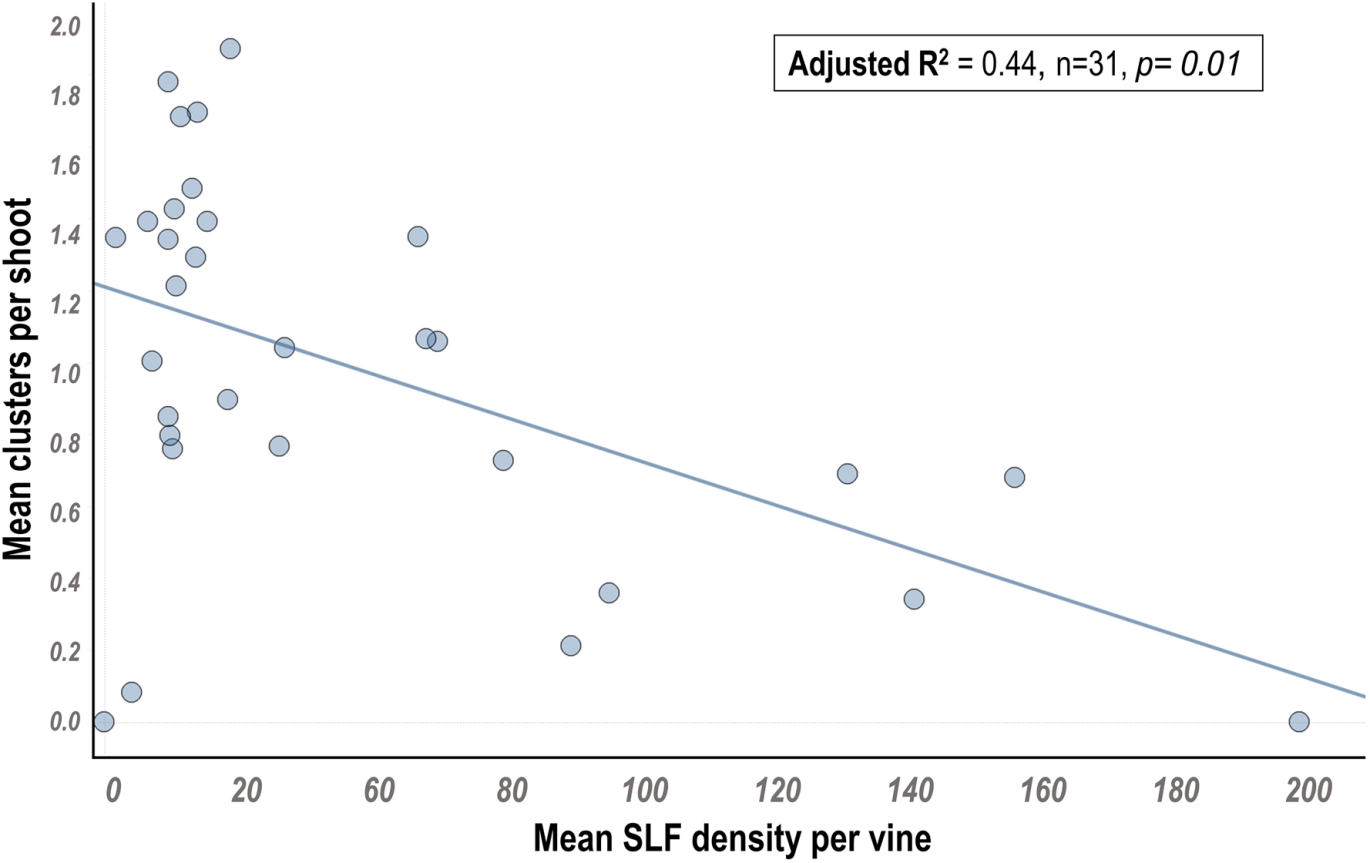
Relationship between mean spotted lanternfly (SLF) density per vine and grape cluster production (clusters per shoot/vine). Each point represents a single vine that was scouted for the SLF during the growing season. Cluster production on each vine was taken in the subsequent season.

Adult SLF numbers were significantly negatively associated with two plant health metrics: cluster production and leaf redness (cluster production: F = 6.3, df = 1, p = 0.01; leaf redness: F = 6.7, df = 1, p = 0.0009*).* Reduced fruit clusters were associated with high numbers of SLF in the previous growing season (adjusted R^2^ = 0.44) (Fig. 3). Additionally, leaf redness significantly increased with increased SLF infestation (adjusted R^2^ = 0.41, data not shown). Other health metric measures (percentage of dead buds and crown gall severity) were not associated with adult SLF infestation in the previous year (p > 0.05, data not shown).

### Grid sampling

**Figure 4:**
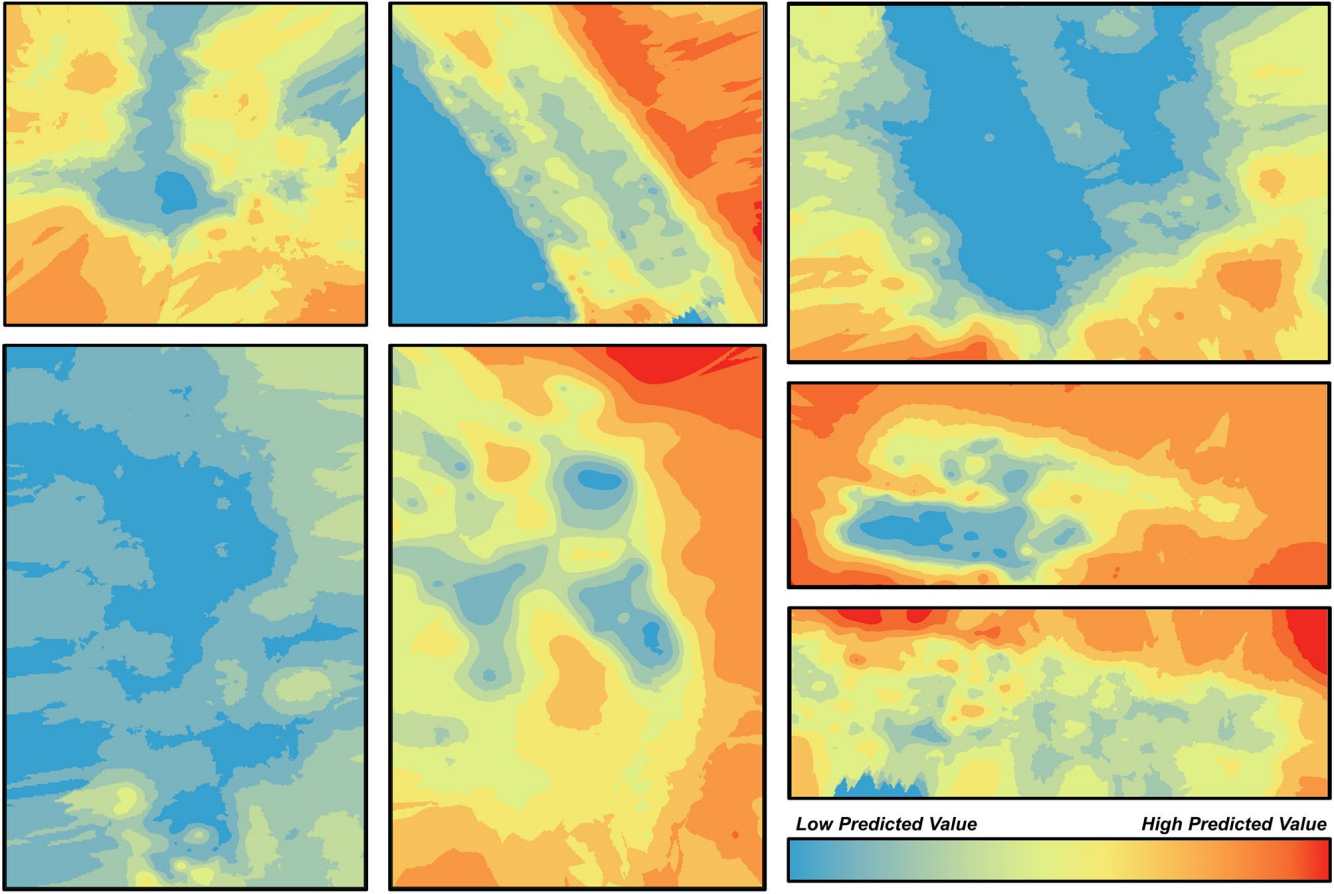
Empirical Bayesian Kriging (EBK) interpolation of adult spotted lanternfly (SLF) densities within vineyard blocks across 7 vineyards. Each square indicates a single vineyard block. Darker (red) colors indicate higher predicted density of SLF egg masses, whereas lighter colors (blue) indicate lower predicted densities.

**Figure 5:**
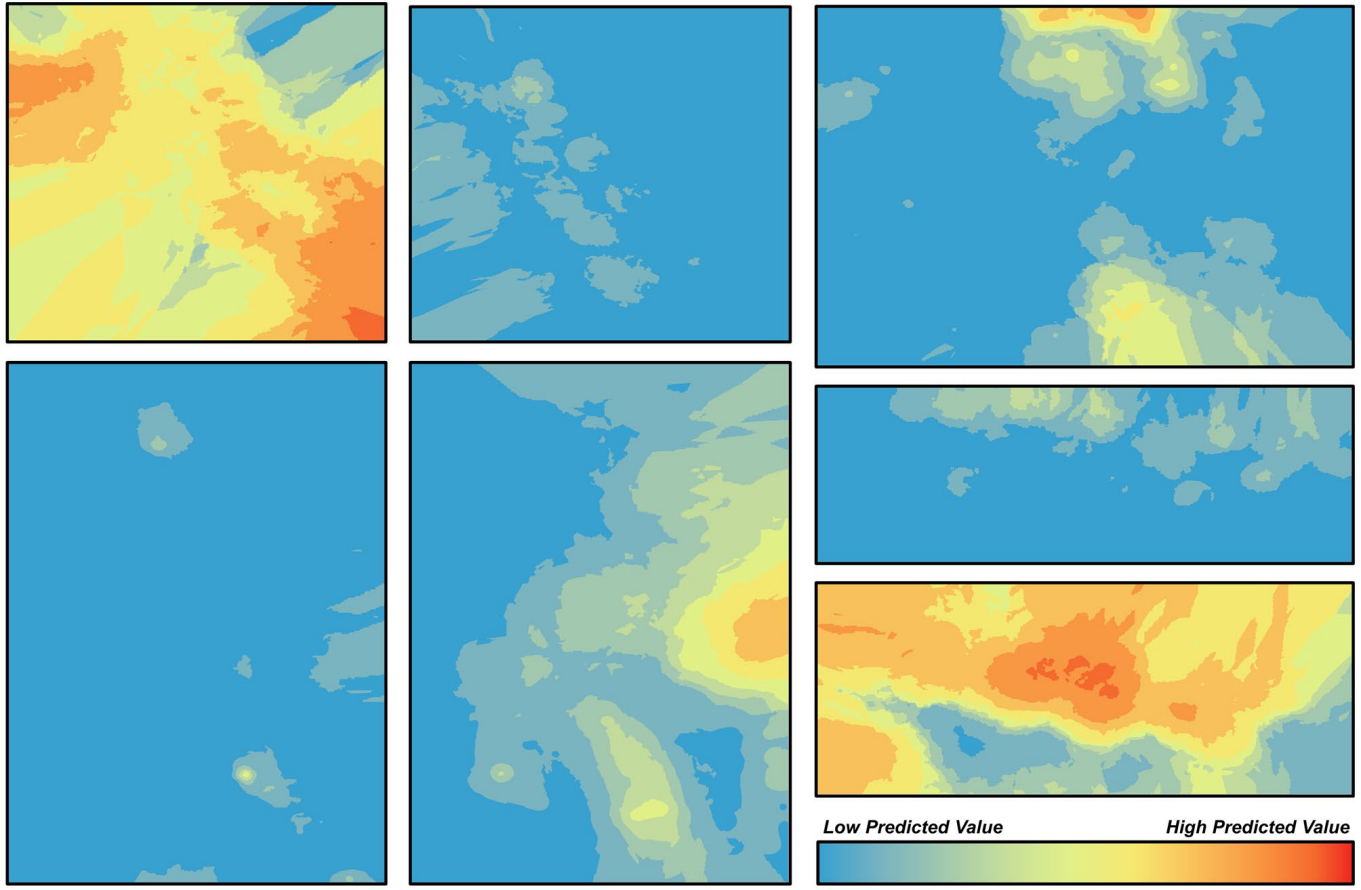
Empirical Bayesian Kriging (EBK) interpolation of spotted lanternfly (SLF) egg mass densities within vineyard blocks across 7 vineyards. Each square indicates a single vineyard block. Darker (red) colors indicate higher predicted density of SLF adults, whereas lighter colors (blue) indicate lower predicted densities.

Distance from the wood edge was a significant factor in total adult abundance (χ^2^ = 16.6, df = 1, p < 0.0001). On average, 2 times more adult SLF were found within the first 15 m of the vineyard edges compared to the interior of the vineyard. Distance from the wood edge also significantly influenced egg mass abundance (χ^2^ = 181.8, df = 1, p < 0.0001). On average, 2.8 times more SLF egg masses were found within the 15 m of the vineyard edges compared to the interior of the vineyard block. SLF across all vineyards were significantly spatially autocorrelated using Moran’s I statistic with the Moran’s index ranging from 0.19 to 0.68 (Supplemental Table S1). All egg mass distributions were significantly spatially autocorrelated except for one location which was not significant (Vineyard G, indicating spatial randomness). Moran’s index values were generally lower for egg masses, ranging from 0.05 to 0.3. Mapping results from Empirical Bayesian Kriging (EBK) show high predicted values on the outside edge of every vineyard sampled for adults, and low predicted values in the interior of the vineyard (Fig. 4). These results are also shown with egg masses (Fig. 5), but to a weaker extent.

### SLF mark and recapture

**Figure 6:**
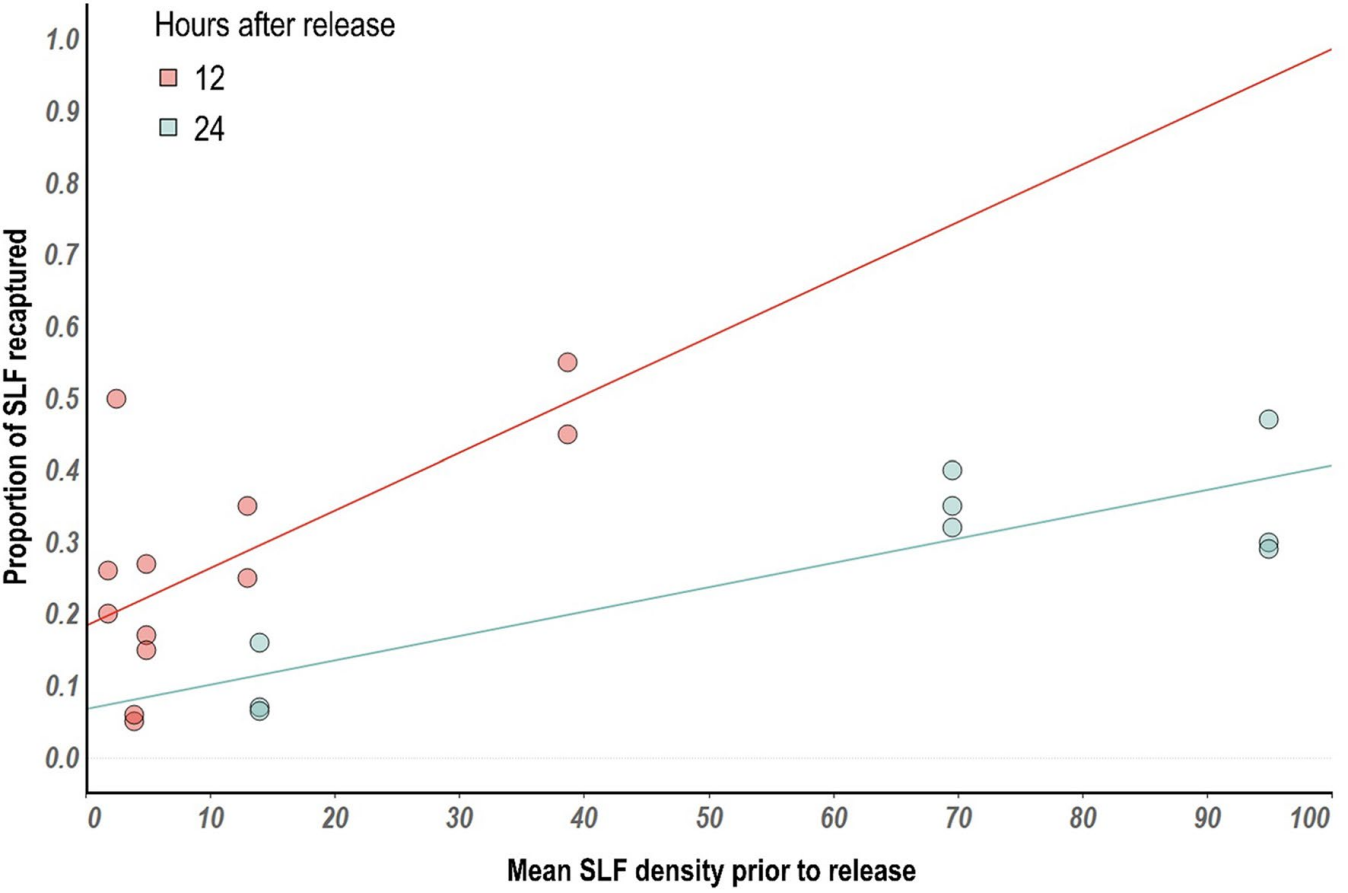
Relationship between mean spotted lanternfly density and proportion of SLF recapture after either 12 or 24 h.

Pre-existing SLF populations on vines significantly impacted the percent of SLF recaptured after both 12 and 24 h (Fig. 6). Vines with greater SLF densities (> 40 SLF/vine) had higher recapture rates than those with lower levels of pre-existing SLF suggesting local retention where more SLF was already present (adjusted R^2^ = 0.49 at 12 h, adjusted R^2^ = 0.66 at 24 h). These results were consistent at both 12 and 24 h (12 h: *F* = 6.5*, df* = 1*, p* = 0.01; 24 h: *F* = 6.7, *df* = *1, p* = 0.009).

## Discussion

SLF is a noted pest of vineyards in the Northeastern U.S., however, little is known about its biology, behavior, and management in these agroecosystems. In this study, we evaluated the spatial and temporal relationships of SLF in grape systems. In line with our proposed hypothesis, we found that the greatest proportions of SLF adults and egg masses were consistently observed along the vineyard perimeters. Further, greater densities of SLF were significantly related to reduced vine health (reduced fruit production and increased leaf redness). This is the first study to report on the spatial organization of SLF within a landscape as well as indicate the potential negative effects of SLF on grapevine yield.

Weekly monitoring and grid sampling of SLF in vineyard blocks revealed clumping of SLF around vineyard edges. From geostatistical modeling using the EBK approach, higher densities of adult SLF were predicted at the outside edge of vineyard blocks and low densities in the vineyard interior. Importantly, this occurs regardless of overall population density within the vineyard. These edge effects are likely impacted by landscape features and the ability of SLF to utilize resources in those landscapes. SLF is not confined to vineyards and is known to disperse into vineyards from wooded edges^15^. Therefore, these relationships are likely significantly impacted by the quantity and quality of nearby host plants. Indeed, in this study we found that vineyard distance from wooded edges was strongly correlated with both SLF adult and egg abundance in the vineyard. Successful SLF control will likely necessitate some type of landscape management, either at a local or regional scale. Edge effects have been observed for other agricultural insect pests (see review^24^) with predictive models that attempt to explain such behavior^25^, which presents an opportunity to exploit this spatial organization for precision pest management. Further research may also address specific landscape factors that explain the prevalence of SLF in certain ecosystems, including land cover, which was not analyzed in this study.

All vineyards monitored in this study used repeated insecticide applications to manage SLF which could alter their spatial organization. Insecticides can be highly efficacious against SLF^18^, thus patterns observed in this study may be a result of large daily influxes to vineyards^15^. Insecticide exposure could also cause sublethal effects on SLF (e.g. reduced mobility). Therefore, it is possible that SLF may assume a different spatial distribution in unmanaged or infrequently sprayed vineyards. While egg masses displayed a clumped distribution at vineyard edges, this pattern had a higher degree of variance compared to adult distributions. Further study is needed to elucidate the biological and/or ecological mechanisms that explain this discrepancy. Our previous study indicated that SLF densities decrease in vineyards towards the end of the growing season^15^ which may reduce overall ovipositional activity. Female SLF may prefer to oviposit in nearby wooded edges, thereby reducing egg laying in vineyards and decreasing edge effects associated with egg masses.

Our findings suggest that insecticide regimes can be optimized to control SLF populations. Specifically, vineyard edges infested with SLF can be targeted with insecticidal sprays (e.g. perimeter sprays) which will reduce overall insecticide use and cost and mitigate negative off-target effects. Furthermore, management that focuses on the reducing SLF populations at vineyard edges may prevent infestations in the vineyard interior. Non-chemical approaches, like netting along vineyard edges, may limit SLF dispersing into vineyards. These tactics have been successful with highly mobile insects including brown marmorated stink bug^26^. Other vineyard pests, such as grape berry moth and Japanese beetle also exhibit edge effects and may increase the utility of these targeted management efforts (e.g. perimeter sprays) in grape production^27^.

The number of clusters per shoot were negatively associated with increased adult SLF from the prior year. Primary buds, which bear the majority of fruit, are the most sensitive to cold damage, followed by secondary buds (reduced fruit) and tertiary buds (no fruit)^28^. If SLF feeding reduces overwintering resources for the vine, this could manifest as increased damage to primary buds rather than complete bud death. This may explain why our study failed to find a significant effect of SLF abundance on the prevalence of dead shoots. Red leaves on red producing cultivars were significantly related with SLF abundance, which could be used as a metric to predict stressed plants and inform potential changes in sap flow and nutrient status of the vine^29^. Crown gall was not significantly related to SLF abundance; however, our study did not include measurements of crown gall in the vineyards prior to SLF infestations, which limits our ability to address this relationship. Further study is needed to evaluate the relationship between SLF feeding injury and the severity and spread of crown gall. Additionally, nymphal SLF were not evaluated in this study and may influence these vine health metrics, although low levels of this life stage have been documented in vineyards^15^.

Our mark and recapture results suggest that SLF may aggregate in areas with higher densities of other SLF individuals. Thus, the relationships presented here are likely not only a result of vineyard management but also SLF behavior. Advantages of feeding in groups including overcoming plant defenses or mate selection may explain these patterns. These results should be further explored as they could play a critical role in developing mass-trapping, monitoring, or behavioral disruption tactics. It is important to note that the potential effects of powder-marked SLF on their mobility has not been fully evaluated and this should be studied in greater detail. Furthermore, this study only examined the short-term retention of SLF within an area (12–24 h), and longer time periods (7–14 days) may provide meaningful insights to behavior and management of SLF.

The spatial concentration of SLF adults at vineyard edges was consistent temporally, both between years and weeks of sampling. These findings suggest a predictable trend in behavior which can be used to optimize monitoring and precise targeting of management efforts, including release of natural enemies and chemical applications in these agroecosystems. Utilizing this knowledge to modify chemical applications could reduce overall insecticide input into the production system, reducing non-target effects and secondary pest problems.

## Methods

### Weekly monitoring

SLF infestations were sampled weekly in vineyards within southeastern Pennsylvania in 2018 and 2019. Six vineyards were used in both 2018 and 2019, plus an additional 2 vineyards were added in 2019. Weekly monitoring for adult SLF began in September in 2018 and August in 2019. In both years, monitoring continued until no more SLF were observed in each vineyard. At least two rows were randomly selected within each vineyard block and the number of SLF were recorded every 15 m along a transect spanning the entire length of the row. The length of each row varied depending on the vineyard (50 m–210 m). At each distance, the total number of SLF on the vine were counted. SLF was monitored on *Vitis vinifera* varieties in all vineyards, and a subset of vineyards (n = 4) also included monitoring on hybrid *Vitis* cultivars. Vineyards were actively managed for SLF and other pests with insecticide applications.

### Vine health

In grapevines, the fruit set in a given year is determined in the year prior, when buds are being formed. After overwintering, these buds provide shoots and clusters for the following season. In spring 2019 and 2020, the number of dead shoots, living shoots, and clusters on each shoot were counted on all monitored vines to evaluate the relationship between SLF infestation and bud fruitfulness and survival. Leaf redness in grapevines has been related to reduced translocation and accumulation of sugars within the canopy^29^, which may be linked to phloem feeding by SLF. In the fall of 2019, percent leaf redness was estimated on a scale of 0–100% for all monitored vines. Leaf redness was only observed on red fruiting cultivars (e.g. ‘Pinot Noir’, ‘Chambourcin’). Similarly, crown gall severity at the trunks and cordon were taken for each vine due to the association of crown gall (caused by the bacterium *Agrobacterium vitis*) and grapevine decline, which can be exacerbated by physical injury to the plant^30^. Severity was ranked on a scale of 0 (no crown gall visible) to 5 (extreme crown gall). For analysis, the severity rankings for both cordon and trunk were combined.

### Grid sampling

Adult and egg mass counts were completed in selected blocks within 7 different vineyards in southeastern Pennsylvania. Vineyard block size varied with the smallest being 0.4 hectare and the largest 1.5 hectares. Blocks were often mixed cultivar plantings and varied in SLF infestation pressure. Adults were sampled between 16 and 27 Sept 2019 and egg masses were sampled from 7 to 16 Oct 2019. Within each block, vines were selected every 5 m along each row and the total number of SLF adults and egg masses counted.

### SLF mark and recapture

Adult SLF (n = 348) were collected from unmanaged forested areas and marked with fluorescent powder (DayGlo Color Corp., Cleveland, OH). SLF were randomly split into groups of 20 and released within vineyard rows featuring variable pre-existing densities of SLF. Prior to the release of the marked SLF adults, densities of SLF in experimental vineyard rows were determined by counting a subset of vines in each row. Marked SLF were placed at the base of vines in each vineyard row. After either 12 or 24 h, vines were examined, and the number of remaining of marked SLF counted. The powder was tested prior to the initiation of the experiment on adult SLF to ensure it would not lead to increased mortality after 48 h of exposure.

### Data analysis

Weekly monitoring data were analyzed using generalized linear mixed models (GLMM) (R version 3.6.2) (R package; ‘lme4’^31^). The proportion of SLF at each distance was determined from the total number of SLF counted in that row. Proportion of SLF at each distance was analyzed using fixed effects of distance and site, and year, week, and row as random nested effects using a binomial distribution. In order to determine the effect of vineyard edges on the proportion of SLF, a ‘distance’ variable was assigned to each sampling point within a vineyard transect to indicate the distance from the edge of the vineyard block. Additional analyses tested the effect of vineyard block size and grape variety as fixed effects. Due to the potential variation of grape variety across the different vineyards, only vineyard blocks that contained both *V. vinifera* and hybrid varieties (4/8 vineyards) were included in the analysis with grape variety.

Vine health metrics were assessed using generalized linear mixed models, with either a binomial (leaf redness, shoot death) or a normal distribution (number of clusters, gall severity). Health metrics were modeled with previous SLF infestation (average adult SLF per vine) as the fixed effect whereas vineyard and year were considered random effects in model. Mark and recapture data were analyzed assuming a binomial model where the total number of recaptured SLF was compared to the total number of SLF released. Adjusted R squared values were computed for all significant models using R package, MuMIn^32^. All models were tested for appropriateness of fit by testing for overdispersion using package blmeco and function dispersion_glmer()^33^ or normality using functions qqnorm() and qqline().

Grid sampling data was georeferenced with latitude and longitude using Google Earth. Using ArcGIS Pro, a polyline was drawn around the wood edge for each vineyard, and the distance of each grid sampling point was then calculated to the nearest wood edge (‘Near’ tool, ArcGIS Pro version 2.5.0). The distance from the wooded edge and the number of SLF (adults and egg masses) were then analyzed using a GLMM with row and vineyard as random nested effects using a Poisson distribution in R. The number and position of SLF in each vineyard were also analyzed in ArcGIS Pro using Anselin Local Moran’s I tool to determine spatial relatedness of points^34,35^. A Moran’s I Index value of 1 signifies strong spatial autocorrelation (clustering), 0 indicates no spatial autocorrelation (random), and -1 indicates highly dispersed autocorrelation. Empirical Bayesian Kriging (EBK) was also used to observe spatial trends in abundance of egg masses and adult SLF within each vineyard block. EBK is a mapping method to predict unmeasured values using measured values within a spatial framework^36^. For all EBK models, the power function was used to create the semiovariograms.

## Supplementary information

Supplementary Information.

## References

[1] Prasad AM, Iverson LR, Peters MP, Bossenbroek JM, Matthews SN, Sydnor TD, Schwartz MW (2010). Modeling the invasive emerald ash borer risk of spread using a spatially explicit cellular model. Landsc. Ecol..

[2] Huang D, Zhang R, Kim KC, Suarez AV (2012). Spatial pattern and determinants of the first detection locations of invasive alien species in mainland China. PLoS ONE.

[3] Roy HE, Adriaens T, Isaac NJ, Kenis M, Onkelinx T, Martin GS (2012). Invasive alien predator causes rapid declines of native European ladybirds. Divers. Distrib..

[4] Cini A, Anfora G, Escudero-Colomar LA, Grassi A, Santosuosso U, Seljak G, Papini A (2014). Tracking the invasion of the alien fruit pest *Drosophila suzukii* in Europe. J. Pest Sci..

[5] Filipe AF, Quaglietta L, Ferreira M, Magalhães MF, Beja P (2017). Geostatistical distribution modelling of two invasive crayfish across dendritic stream networks. Biol. Invas..

[6] Hahn NG, Rodriguez-Saona C, Hamilton GC (2017). Characterizing the spatial distribution of brown marmorated stink bug, *Halyomorpha halys* Stål (Hemiptera: Pentatomidae), populations in peach orchards. PLoS ONE.

[7] Carrière Y, Goodell PB, Ellers-Kirk C, Larocque G, Dutilleul P, Naranjo SE, Ellsworth PC (2012). Effects of local and landscape factors on population dynamics of a cotton pest. PLoS ONE.

[8] Wang XG, Kaçar G, Biondi A, Daane KM (2016). Foraging efficiency and outcomes of interactions of two pupal parasitoids attacking the invasive spotted wing drosophila. Biol. Control.

[9] Dara SK, Barringer L, Arthurs SP (2015). *Lycorma delicatula* (Hemiptera: Fulgoridae): a new invasive pest in the United States. J. Integr. Pest Manage..

[10] Kim H, Kim M, Kwon DH, Park S, Lee Y, Huang J (2013). Molecular comparison of *Lycorma delicatula* (Hemiptera: Fulgoridae) isolates in Korea, China, and Japan. J. Asia-Pac. Entomol..

[11] Han JM, Kim H, Lim EJ, Lee S, Kwon YJ, Cho S (2008). *Lycorma delicatula* (Hemiptera: Auchenorrhyncha: Fulgoridae: Aphaeninae) finally, but suddenly arrived in Korea. Entomol. Res..

[12] Wakie TT, Neven LG, Yee WL, Lu Z (2019). The establishment risk of *Lycorma delicatula* (Hemiptera: Fulgoridae) in the United States and globally. J. Econ. Entomol..

[13] Jung JM, Jung S, Byeon D, Lee WH (2017). Model-based prediction of potential distribution of the invasive insect pest, spotted lanternfly *Lycorma delicatula* (Hemiptera: Fulgoridae), by using CLIMEX. J. Asia-Pac. Biodivers..

[14] Harper JK, Stone W, Kelsey TW, Kime LF (2019). Potential economic impact of the spotted lanternfly on agriculture and forestry in Pennsylvania. Control Rural Pennsylvania Rep..

[15] Leach H, Leach A (2020). Seasonal phenology and activity of spotted lanternfly (*Lycorma delicatula)* in eastern US vineyards. J. Pest Sci..

[16] Barringer LE, Smyers E (2016). Predation of the spotted lanternfly, *Lycorma delicatula* (White) (Hemiptera: Fulgoridae) by two native Hemiptera. Entomol. News.

[17] Cooperband MF, Mack R, Spichiger SE (2018). Chipping to destroy egg masses of the spotted lanternfly, *Lycorma delicatula* (Hemiptera: Fulgoridae). J. Insect Sci..

[18] Leach H, Biddinger DJ, Krawczyk G, Smyer SE, Urban JM (2019). Evaluation of insecticides for control of the spotted lanternfly, *Lycorma delicatula*, (Hemiptera: Fulgoridae), a new pest of fruit in the Northeastern US. Crop Prot..

[19] Clifton EH, Castrillo LA, Gryganskyi A, Hajek AE (2019). A pair of native fungal pathogens drives decline of a new invasive herbivore. Proc. Natl. Acad. Sci..

[20] Urban JM (2020). Perspective: shedding light on spotted lanternfly impacts in the USA. Pest Manage. Sci..

[21] Wolfin MS, Binyameen M, Wang Y, Urban JM, Roberts DC, Baker TC (2019). Flight dispersal capabilities of female spotted lanternflies (*Lycorma delicatula*) related to size and mating status. J. Insect Behav..

[22] Clifton EH, Hajek AE, Jenkins NE, Roush RT, Rost JP, Biddinger DJ (2020). Applications of *Beauveria bassiana* (Hypocreales: Cordycipitaceae) to control populations of spotted lanternfly (Hemiptera: Fulgoridae), in semi-natural landscapes and on grapevines. Environ. Entomol..

[23] Francese JA, Cooperband MF, Murman KM, Cannon SL, Booth EG, Devine SM, Wallace MS (2020). Developing traps for the spotted lanternfly, *Lycorma delicatula* (Hemiptera: Fulgoridae). Environ. Entomol..

[24] Nguyen HDD, Nansen C (2018). Edge-biased distributions of insects. A review. Agron. Sustain. Dev..

[25] Ries L, Sisk TD (2004). A predictive model of edge effects. Ecology.

[26] Leskey TC, Short BD, Ludwick D (2020). Comparison and refinement of integrated pest management tactics for *Halyomorpha halys* (Hemiptera: Pentatomidae) management in apple orchards. J. Econ. Entomol..

[27] Mason KS, Roubos CR, Teixeira LA, Isaacs R (2016). Spatially targeted applications of reduced-risk insecticides for economical control of grape berry moth, *Paralobesia viteana* (Lepidoptera: Tortricidae). J. Econ. Entomol..

[28] Goffinet, M.C. Anatomy of grapevine winter injury and recovery. Dept. Hort. Services Res. Paper, Cornell Univ. (2004). Accessed April 20, 2020 from https://www.eaglegrapegrowers.org/uploads/1/1/8/4/118472897/anatomy_of_winter_injury_hi_res.pdf.

[29] Halldorson MM, Keller M (2018). Grapevine leafroll disease alters leaf physiology but has little effect on plant cold hardiness. Planta.

[30] Süle S, Burr TJ (1998). The effect of resistance of rootstocks to crown gall (*Agrobacterium* spp.) on the susceptibility of scions in grape vine cultivars. Plant Pathol..

[31] Bates, D., Maechler, M., Bolker, B., Walker, S., Christensen, R.H.B., Singmann, H., Dai, B., Scheipl, F., Grothendieck, G. & Green, P. *R Package ‘lme4’*. 1.17 (2018).

[32] Bartoń, K. *MuMIn: Multi-model Inference. R Package version 1.40.4* (2018).

[33] Korner-Nievergelt F, Roth T, von Felten S, Guelat J, Almasi B, Korner-Nievergelt P (2015). Bayesian Data Analysis in Ecology Using Linear Models with R, BUGS and Stan.

[34] Anselin L (1995). Local indicators of spatial association—LISA. Geogr. Anal..

[35] Mitchell A (2005). The ESRI Guide to GIS Analysis.

[36] Krivoruchko K (2011). Spatial Statistical Data Analysis for GIS Users.

