# Supplementary material for: Characterizing the spatial distributions of spotted lanternfly (Hemiptera: Fulgoridae) in Pennsylvania vineyards
Source: Sci Rep. 2020 Nov 25;10:20588. doi: 10.1038/s41598-020-77461-9 (PMC7688957; doi:10.1038/s41598-020-77461-9)
Supplement: Supplementary file 1 — Supplementary Information. [file 41598_2020_77461_MOESM1_ESM.docx]

**SUPPLEMENTAL FIGURES**

**Characterizing the spatial distributions of spotted lanternfly (Hemiptera: Fulgoridae) in Pennsylvania vineyards**

Authors: Ashley Leach^1^ and Heather Leach^2*^

**SUPPLEMENTAL FIGURES**


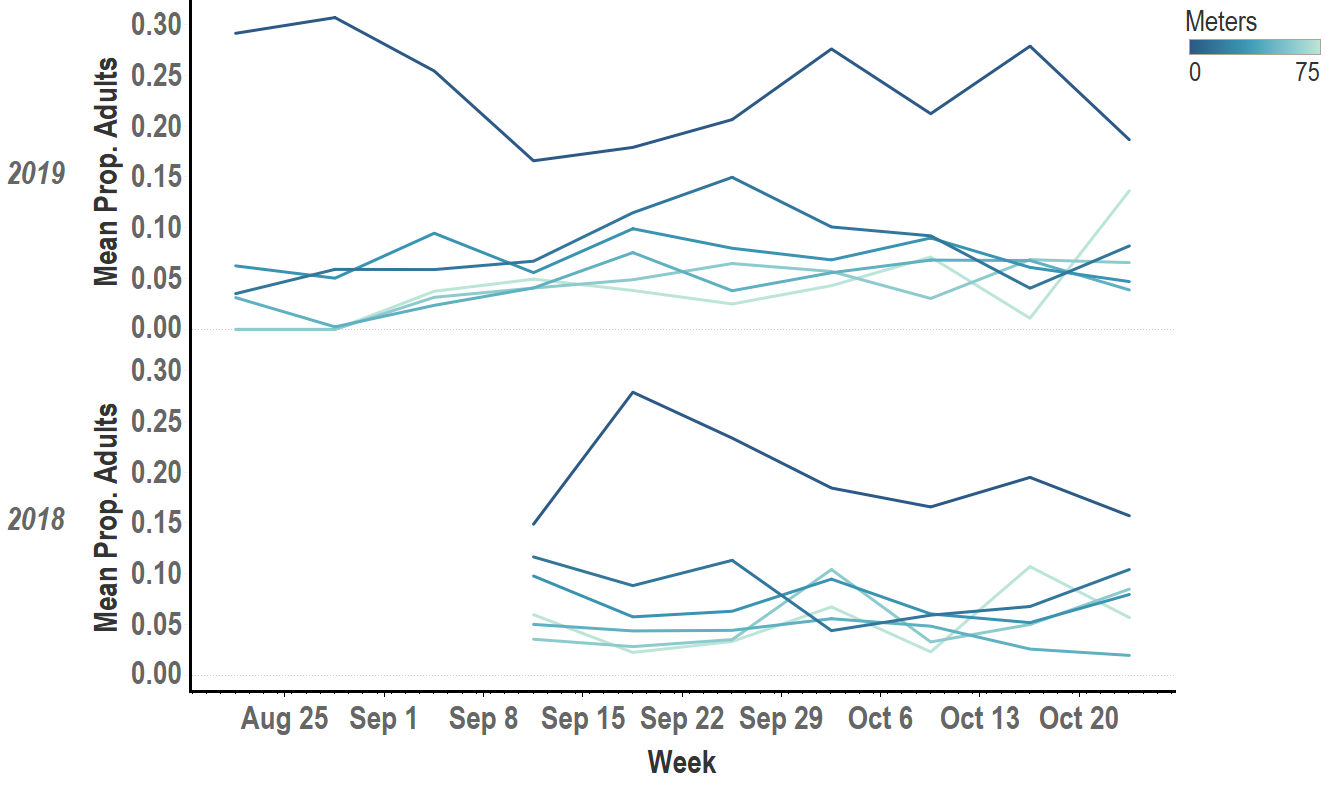


**Supplemental figure 1:** Mean weekly proportion adult SLF found at sampling distances from vineyard edge, 0 m (at vineyard edge) to 75 m. Vineyards were sampled for SLF during 2018 (7 weeks, 6 vineyards) and 2019 (10 weeks, 8 vineyards). Scouting was initiated at first appearance of adults in vineyards and continued until no more SLF were observed.


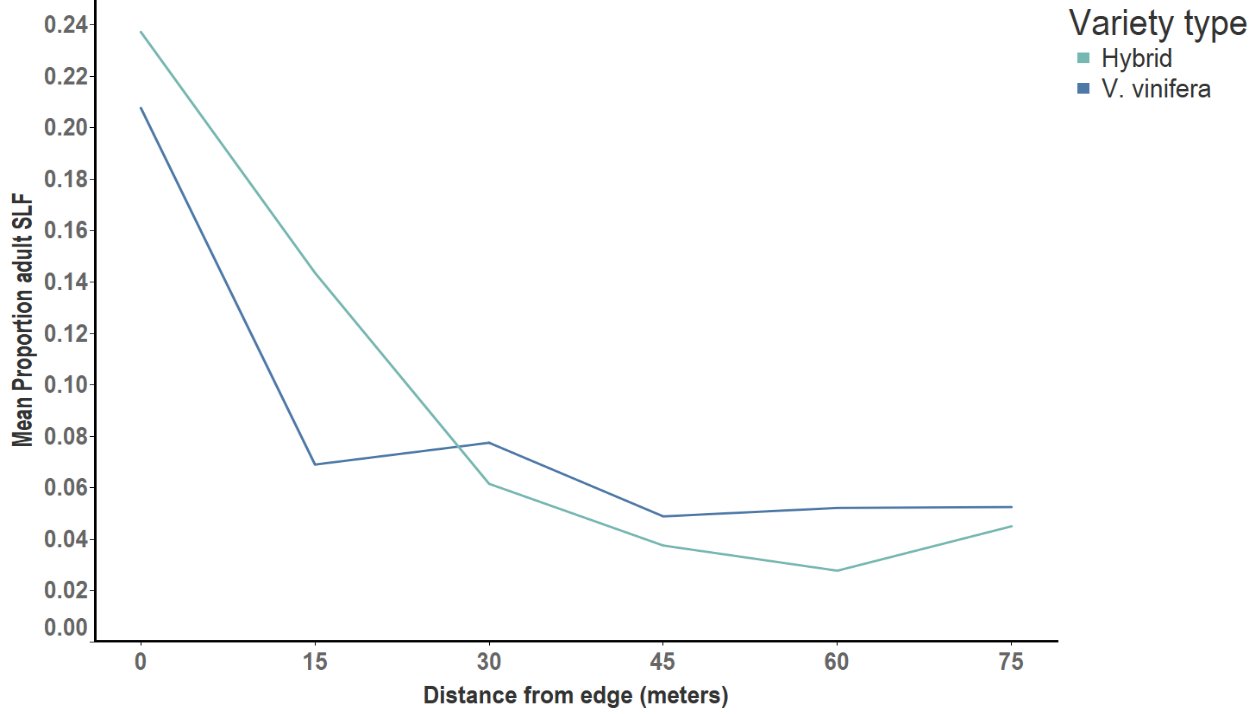


***a)***

***b)***


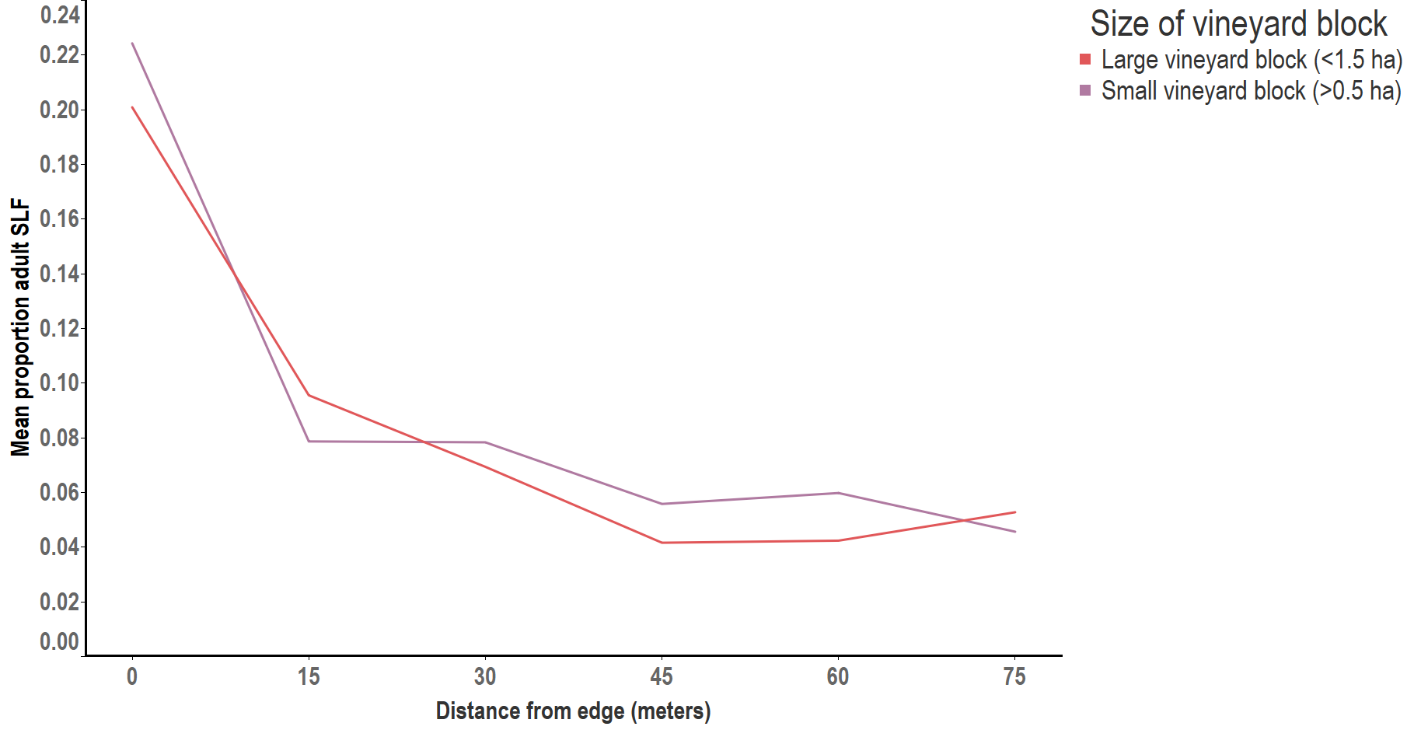


**Supplemental figure 2:** A) Mean proportion adult SLF found at sampling distances from vineyard edge, 0 m (at vineyard edge) to 75 m in either large (<1.5 ha) vineyard blocks or small (>0.5 ha) vineyard blocks. B) Mean proportion adult SLF found at sampling distances from vineyard edge, 0 m (at vineyard edge) to 75 m in two varieties, *Vitis vinifera* and hybrid *Vitis* species. A total of 6 vineyards were sampled for SLF during 2018 and 8 vineyards in 2019.

| **Supplemental Table 1.** The Anselin Local Moran’s I index scores and p-values for spotted lanternfly adults and egg masses across all sampled vineyards. The Moran’s I index determines spatial relatedness of points. A Moran’s I Index value of 1 signifies strong spatial autocorrelation (clustering), 0 indicates no spatial autocorrelation (random), and -1 indicates highly dispersed autocorrelation. Significant p-values (alpha = 0.05) are bolded. | | |
| --- | --- | --- |
| **Vineyard** | **Moran’s Index** | **P-Value** |
| **Adult spotted lanternfly** | | |
| Vineyard A | 0.46 | **< 0.0001** |
| Vineyard B | 0.16 | **< 0.0001** |
| Vineyard C | 0.32 | **< 0.0001** |
| Vineyard D | 0.20 | **< 0.0001** |
| Vineyard E | 0.43 | **< 0.0001** |
| Vineyard F | 0.59 | **< 0.0001** |
| Vineyard G | 0.69 | **< 0.0001** |
| **Spotted lanternfly egg masses** | | |
| Vineyard A | 0.30 | **< 0.0001** |
| Vineyard B | 0.07 | **0.01** |
| Vineyard C | 0.23 | **0.0008** |
| Vineyard D | 0.11 | **0.0003** |
| Vineyard E | 0.13 | **0.01** |
| Vineyard F | 0.11 | **< 0.0001** |
| Vineyard G | 0.05 | 0.09 |
